# Efficient skin interactions of graphene derivatives: challenge, opportunity or both?†

**DOI:** 10.1039/d3na00574g

**Published:** 2023-10-11

**Authors:** Fatemeh Zabihi, Zhaoxu Tu, Sabine Kaessmeyer, Fabian Schumacher, Fiorenza Rancan, Burkhard Kleuser, Christoph Boettcher, Kai Ludwig, Johanna Plendl, Sarah Hedtrich, Annika Vogt, Rainer Haag

**Affiliations:** a Institut für Chemie und Biochemie, Freie Universität Berlin Takustr. 3 Berlin 14195 Germany fatemezabihi@zedat.fu-berlin.de haag@chemie.fu-berlin.de +49-030-8385-2633; b Department of Dermatology and Allergy, Clinical Research Center for Hair and Skin Science, Charité Universitaetsmedizin Berlin Germany; c The Sixth Affiliated Hospital of Sun Yat-sen University Guangzhou Guangdong China; d Department of Veterinary Medicine, Institute of Veterinary Anatomy, Freie Universität Berlin Germany; e Institute of Pharmacy (Pharmacology and Toxicology), Freie Universität Berlin 14195 Berlin Germany; f Forschungszentrum für Elektronenmikroskopie, Institut für Chemie und Biochemie, Freie Universität Berlin Fabeckstr. 36a 14195 Berlin Germany; g Faculty of Pharmaceutical Sciences, University of British Columbia 2405 Wesbrook Mall V6T1Z3 Vancouver Canada; h Berlin Institute of Health at Charité, Universitaetsmedizin Berlin Lindenberger Weg 80 13125 Berlin Germany; i Division of Veterinary Anatomy, Vetsuisse Faculty, University of Bern 3012 Bern Switzerland

## Abstract

Interactions between graphene, with its wide deployment in consumer products, and skin, the body's largest organ and first barrier, are highly relevant with respect to toxicology and dermal delivery. In this work, interaction of polyglycerol-functionalized graphene sheets, with 200 nm average lateral size and different surface charges, and human skin was studied and their potential as topical delivery systems were investigated. While neutral graphene sheets showed no significant skin interaction, their positively and negatively charged counterparts interacted with the skin, remaining in the stratum corneum. This efficient skin interaction bears a warning but also suggests a new topical drug delivery strategy based on the sheets' high loading capacity and photothermal property. Therefore, the immunosuppressive drug tacrolimus was loaded onto positively and negatively charged graphene sheets, and its release measured with and without laser irradiation using liquid chromatography tandem-mass spectrometry. Laser irradiation accelerated the release of tacrolimus, due to the photothermal property of graphene sheets. In addition, graphene sheets with positive and negative surface charges were loaded with Nile red, and their ability to deliver this cargo through the skin was investigated. Graphene sheets with positive surface charge were more efficient than the negatively charged ones in enhancing Nile red penetration into the skin.

## Abstract 2

Positively and negatively charged graphene sheets interacted with human skin and enhanced penetration of Nile red-loaded into the epidermal and dermal layers of the skin. Moreover, graphene's cargo was released more efficiently into the skin layers after laser exposure.

## Graphical abstract

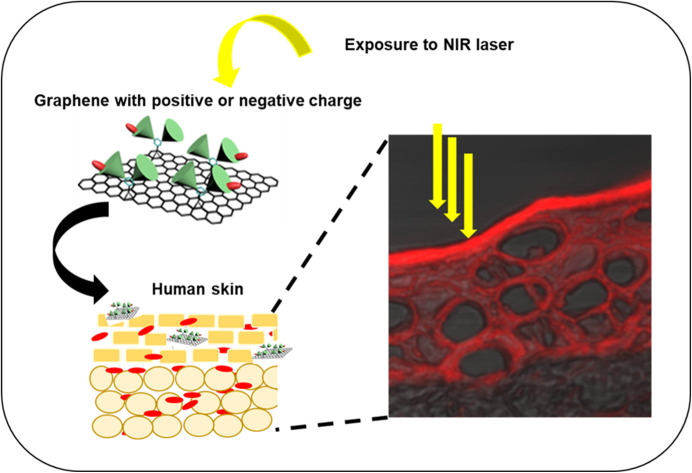

## Introduction

As the waste products of graphene derivatives are discharged in greater amounts, due to their production in larger scales for a growing range of applications, the health risks of occupational and environmental exposure to these materials grow in importance.^1–3^ Several main factors influence graphene derivatives' biointeractions and long-term toxicity inside the body: the route of their internalization into the body (respiratory, oral, ocular or cutaneous), along with their physicochemical properties, including size, charge and hydrophilicity.^4–11^ To date, most toxicity studies have sought to assess the adverse effects of graphene derivatives on the respiratory system.^12^ Graphene oxide, as the most hydrophilic and layered graphene derivative, has shown the highest pulmonary toxicity.^12–16^ Along with these comprehensive studies, other reports have demonstrated that the functionality and surface charge of graphene derivatives strongly influence their toxicity and interactions with the body's organs.^9,17,18^ Surface functional groups determine the charge and polydispersity of graphene sheets, and thus dominate their layered structures and aggregations in physiological mediums, which are strongly correlated with the materials' toxicity.^19–22^ Functionalization of graphene derivatives by polymers and biomacromolecules improves their dispersibility in aqueous solutions and dramatically changes their biodistribution, toxicity and cellular interactions.^23–25^ Graphene sheets of few layers, functionalized by poly-ethylene glycol, have shown high uptake and retention in the lung, spleen, liver and kidney without significant accumulation in the brain or the heart.^26^ Polyethylene glycol-functionalized graphene sheets have induced different types of damage, including congestion, necrosis, fibrosis, and glomerular filtration dysfunction, as well as major alterations to gene expression profiles in the organs mentioned above. It has also been shown that passivation of graphene oxide by polyethylene glycol stimulates potent cytokine responses in peritoneal macrophages.^27^ In spite of such negative results and high toxicity, other reports showed no significant toxicity for polyethylene glycol-functionalized graphene sheets, with efficient renal and fecal excretion. Such contradictory results may be due to differences in physiochemical properties of the studied graphene sheets, including size, functionality and surface charge.^28^ Skin, as the largest and first body's organ is at high risk upon exposing to graphene derivatives. However, our knowledge regarding interactions between skin and graphene derivatives mostly is limited to *in vitro* studies including interactions with skin fibroblasts,^29^ keratinocytes and human epidermis.^30–32^ It has been shown that exposing of HaCaT skin keratinocytes to few layers of graphene derivatives for a short time results in reversible toxicity, *i.e.*, these cells were able to recover from the toxic effects. Moreover, it has been demonstrated that graphene derivatives exfoliated by surfactants induce skin irritation, while others do not provoke an adverse effect upon single exposure.^33,34^ Considering graphene materials for dermal application, it is essential to address their skin sensitization and irritation as a possible adverse outcome. Recently, an *in vivo* study suggested dermal exposure of graphene oxide (GO) and few-layer graphene (FLG) has no irritation or sensitization effect on skin immune cells.^35^

On a positive side and in the less-toxic concentration range, polymer-functionalized graphene sheets are interesting candidates for biomedical applications due to their high loading capacity, fast cellular uptake, and photothermal property.^36^ They have been used for loading therapeutic agents and targeting these cargos to tumor sites.^37^ Hyperbranched polyglycerol and polyglycerol-based nanomaterials are hydrophilic polyfunctional vectors with low immunogenicity and high potential for future biomedical applications.^38,39^ Passivation of graphene sheets with polyglycerol has resulted in two-dimensional platforms with unique physicochemical properties and high potential for future biomedical applications.^40–44^ Interactions of polyglycerol-functionalized graphene sheets with cells, their cellular uptake pathways, and even their journey inside the cells depend strongly on their functionality, size and surface charge.^45,46^ Considering these factors, polyglycerol-functionalized graphene sheets have been used for various biomedical applications, including pathogen incapacitation and tumor therapy.^43,47^ Moreover, polyglycerol and similar polymeric nanocarriers have been used as topical drug delivery systems abundantly,^39,48,49^ but their main disadvantage is the lack of a stimuli-responsive factor to trigger the release their cargo in the target tissue. Hybridization of polyglycerol with graphene results in new systems, which can respond to laser as a triggering factor. In addition, graphene will improve interactions of polyglycerol derivatives with the biosystems, as it has been observed for single cells.^45,50^

Accordingly, polyglycerol-functionalized graphene sheets can be used as new topical drug delivery systems, owing to their high loading capacity for hydrophobic drugs such as tacrolimus (TAC). Acceleration of the rate of release of their cargo in the target tissue decreases their side effects and increase their therapeutic efficiency. Moreover, they can induce heat in the target layer of skin, resulting in photothermal therapy. Due to such properties, polyglycerol-functionalized graphene sheets can be used for the sustainable delivery of topical drugs. They can enhance the bioavailability, stability and skin penetration of drugs, which in turn increase their therapeutic effects.

**Scheme 1:**
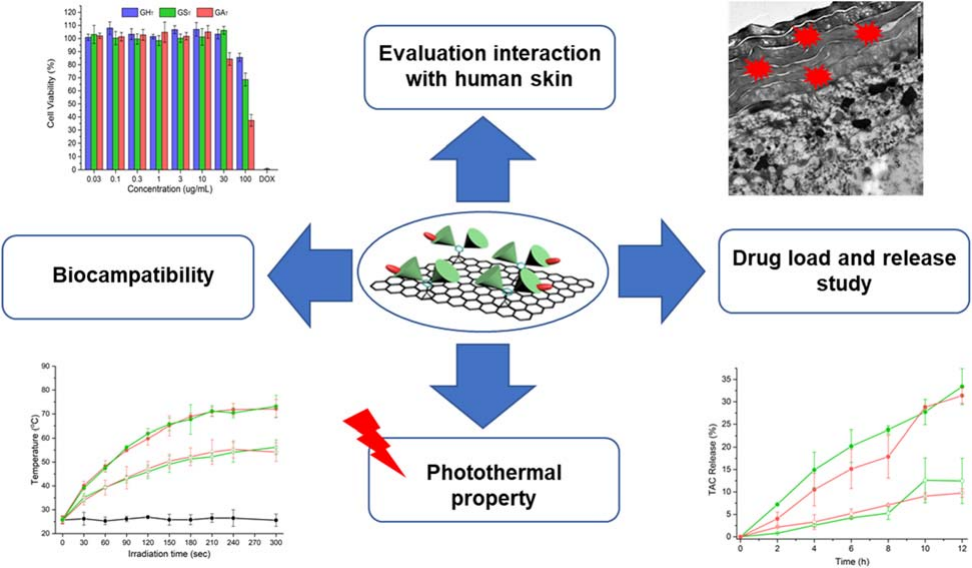
Schematic representation of different experiments in this work.

This work uses microscopic techniques to investigate interactions between human skin and polyglycerol-functionalized graphene sheets with 200 nm lateral size and different surface charges (Scheme 1). While neutral polyglycerol-functionalized graphene sheets showed no significant interaction with human skin, their analogs with negative and positive surface charges interacted efficiently with the skin. Fluorescence microscopy and transmission electron microscopy/tomography images showed that positively charged graphene sheets internalize into the top layer of skin, namely the stratum corneum. These results show that cutaneous exposure to graphene derivatives could result in serious health risks. However, this observation also opens new avenues for the dermal delivery and controlled release of therapeutic agents by graphene derivatives. Accordingly, for the dermal delivery of Nile red we chose polyglycerol-functionalized graphene sheets with positive and negative surface charges that showed significant skin interaction. Fluorescence microscopy images showed that graphene sheets of both positive and negative charge were able to deliver Nile red into both the epidermal and dermal layers of skin. Interestingly, graphene's cargo was released more efficiently into the skin layers under laser irradiation. Our data shows that positively charged graphene sheets, if deployed in a less-toxic concentration range, can be used as efficient dermal delivery systems. Along significant skin uptake, other potential benefits of these materials include high drug-loading capacity (∼50% w/w) for tacrolimus TAC and controlled release of the drug with laser irradiation. However, potential health risks after cutaneous graphene sheet exposure should be considered.

## Experimental

### Materials

Thermally reduced graphene oxide (TRGO) was synthesized according to the method in literature.^51^ Hyperbranched polyglycerol (hPG), with Mn ≈ 10 000 g mol^−1^, was synthesized *via* one-pot, ring-opening anionic polymerization (ROAP).^52^ hPG covered TRGO (hPG-Gs), hPG-amine covered TRGO (hPGN-Gs) and hPG-sulfate covered TRGO (hPGS-Gs) with a size of around 200 nm were prepared according to our previously published articles.^45,46^ IDCC NHS-ester (IDCC-NHS, IDCC is a derivative of Cy5) was bought from IC Discovery GmbH. Cell counting kit-8 (CCK-8) assay, Hoechst 33342 were purchased from Thermo Fisher Scientific. Milli-Q water was used in all experiments. Phosphate-buffered saline (PBS) (10×) pH 7.4 (Thermo Fisher Scientific) was diluted 10 times with Millipore quality water.

### Instrumentation and methods

#### Characterization methods

Atomic Force Microscopy (AFM) was measured with a MultiMode Nanoscope V scanning probe microscopy (SPM) system (Bruker, USA) in air under ambient conditions. Zeta potential data were obtained in PBS solution by Malvern (UK) NANO ZSPO. For transmission electron microscopy (TEM) a 5 μL droplet of sample solution was placed on a hydrophilized carbon film covered copper grid (400 mesh). The supernatant fluid was removed by blotting with a filter paper and the sample was allowed to dry in air. A standard holder was used to transfer the dried samples into a Talos L120C transmission electron microscope (Thermo Fisher Scientific Inc., Waltham, Massachusetts, USA) operated at an acceleration voltage of 120 kV. Micrographs were recorded with a 4k × 4k Ceta 16M camera. The near-infrared (NIR) laser irradiation was performed with a diode infrared laser module (808 nm), Changchun New Industries (China). CCK8 assay was measured with a TECAN (Switzerland) Infinite M200 Pro microplate reader. Confocal laser scanning microscopy (CLSM) was conducted by Leica microscope (Germany) TCS SP8 (63× oil-immersion objective lens). Tacrolimus quantification by stable-isotope dilution liquid chromatography tandem-mass spectrometry (LC-MS/MS) was performed as reported^53^ by means of an Agilent 1260 Infinity LC system coupled to an Agilent 6490 triple quadrupole-mass spectrometer (both from Waldbronn, Germany) interfaced with an electrospray ion source operating in the positive ion mode (ESI+).

### Methods

#### IDCC labeling

hPG-Gs (0.12 g, 1.6 mmol) was dissolved in dry DMF (10 mL). IDCC (0.010 g, 0.24 mmol) was added, and the solution was stirred for 48 h at 60 °C. The solution was cooled to room temperature and dialyzed against deionized water for 48 h to obtain the IDCC – (0.10 g, 84%). IDCC labeling of hPGN-Gs and hPGS-Gs were performed with the same procedure UV/vis (H_2_O): *λ*_max_: 500 nm (1 mg mL^−1^).

#### TAC encapsulation

1 mg hPG-Gs was dispersed in 10 mL PBS. Then 2 mg TAC (dissolved in 0.5 mL ethanol) was added dropwise into the GH dispersion under stirring at room temperature. The mixture was stirred for 1 h before it was centrifuged at 2000 rpm for 5 min. The supernatant was dialysis in Milli-Q for 24 h to get TAC-loaded hPG-Gs and the precipitate was collected for the measurement of loading capacity. The precipitate was dissolved in 10 mL ethanol. Then the ethanol solution was centrifuged at 5000 rpm for 10 min and 1 mL supernatant was extracted. Subsequently, the solvent was evaporated and the dried TAC was dissolved in acetonitrile for HPLC measurement to determinate the TAC concentration. TAC-loaded hPGN-Gs and TAC-loaded hPGS-Gs could be obtained with the same procedure. The loading capacity of hPG-Gs, hPGN-Gs and hPGS-Gs was determined by the HPLC results.

#### Nile red encapsulation

A 60 μL stock solution (0.5 mg mL^−1^) of Nile red in methanol was added to a PBS solution of hPG-Gs, hPGN-Gs and hPGS-Gs dropwise, while mixture was gently stirred (1200 rpm, 25 °C). It was stirred for additional 2 h at 25 °C and then methanol was evaporated. Subsequently, hPG-Gs, hPGN-Gs and hPGS-Gs loaded with Nile red were dried at 50 °C for 1 h. The obtained compound was dissolved in MilliQ water and stirred for additional 10 min at room temperature (25 °C). The mixture was centrifuged for 10 min at 3000*g* and the supernatant was collected and lyophilized. UV/vis (methanol): *λ*_max_: 350 nm (0.001 mg mL^−1^).

#### Skin penetration experiments

To investigate the interaction of graphene derivatives and topical drug delivery efficiency, IDCC-labelled or nile red-loaded (0.001% w/w) hPG-Gs, hPGN-Gs and hPGS-Gs was studied on excised human skin (obtained from plastic surgery with informed consent of healthy donors, after approval by the Ethics Committee of the Freie Universität Berlin, Institute of Pharmacy (Pharmacology and Toxicology ethics vote EA1/081/13 and Charité—Universitätsmedizin Berlin, approval EA1/135/06, renewed in January 2019) and in accordance with the Declaration of Helsinki guidelines. The *ex vivo* experiments was performed using the Franz cell set-up^54^ and conventional base cream containing 0.001% w/w Nile red served as a control. Briefly, human skin was mounted onto static-type Franz cells with the horny layer facing to the air and the dermis having contact with the receptor fluid phosphate buffered saline pH 7.4 (PBS 33.5 °C, skin surface temperature about 32 °C) stirred at 500 rpm. After 30 min, 36 μL of the test formulations and cream was administrated onto the skin surface (finite-dose approach) and incubated for 18 h. Furthermore, after 6 h of incubation time hPGN-Gs and hPGS-Gs samples were irradiated with NIR laser (808 nm, 0.5 W cm^−2^) continuously over 30 min for a total exposure time of 2.5 min and samples were remained for another 12 h. Subsequently, the treated skin areas were punched, embedded in tissue freezing medium and stored at a low temperature (−80 °C). To determine Nile red penetration, the skin was cut vertically into 10 μm thick slices using a cryotome. Subsequently, the slices were subjected to normal and fluorescence light (Nile red: excitation: 560–540 nm, emission: 630–660 nm, Cy5: excitation: 560–540 nm, emission: 630–660 nm).

#### Preparation of skin samples for transmission electron microscopy (TEM)

After application of the graphene sheets according to our previously reported paper,^55^ skin samples were fixed in Karnovsky solution (7.5% glutaraldehyde and 3% paraformaldehyde in 0.1 M cacodylate buffer; all Roth, Karlsruhe, Germany) overnight, washed in 0.1 M cacodylate buffer (cacodylic acid sodium salt trihydrate, Roth, Karlsruhe, Germany), and incubated in 1% osmium tetroxide (Electron Microscopy Sciences, Hatfield, PA, USA) for 4 h. Samples were dehydrated in an ascending series of ethanol followed by the intermedium propylene oxide (1,2-epoxypropan; VWR, Germany). The specimens were subsequently embedded in a mixture of Agar 100 (epoxy resin), DDSA (softener), MNA (hardener) and DMP 30 (catalyst) (all: Agar Scientific; Stansted, UK). Polymerization was done at 45 °C and 55 °C, each for 24 hours. Semi- and ultrathin sections were cut using an Ultramicrotome Reichert Ultracut S (Leica, Wetzlar, Germany). Semi-thin sections (0.5 μm) were stained with modified Richardson solution for 45 seconds on an electric hot plate adjusted to 80 °C. Semithin sections were examined with a light microscope Olympus CX21 (Olympus, Stuttgart, Germany). Next ultrathin sections (80 nm) were mounted on nickel grids (Agar Scientific, Stansted, UK), contrasted with Uranyless EM stain followed by Reynolds lead citrate 3% (both from Delta microscopies, Mauressac, France) and examined with a transmission electron microscope JEM-1400 plus (JEOL Freising, Germany).

#### Electron tomography

Ultrathin sections were mounted on Formvar filmed grids and frozen before the transfer into the transmission electron microscope by the use of a Vitrobot™ Mark IV (Thermo Fisher Scientific, Hillsboro, Oregon; USA). The Vitrobot was operated at 22 °C and at a relative humidity of 100%. The grids were plunged without further treatment into liquid ethane just above its freezing point and transferred in liquid nitrogen. The grids were “clipped” (= inserted in a rigid clip-ring) and transferred under liquid nitrogen into a Talos Arctica™ TEM (Thermo Fisher Scientific) operated at an accelerating voltage of 200 kV and at a nominal magnification of 4300× or 2600×, respectively. Image series in the tilt range of −64°/64° (2° tilt-increments) were recorded at full 4k resolution resulting in a pixel size of 4.91 nm or 8.01 nm, respectively. The defocus value was set to −20 μm. Image stack alignment and 3D reconstruction were performed in the context of the Inspect 3D software V4.3 (Thermo Fisher Scientific). Stacks of 10 image slices from the reconstructed volume were exported for data presentation.

#### Cell viability studies

All cell experiments were conducted according to German genetic engineering laws and German biosafety guidelines in the laboratory (level 1). Dulbecco Modified Eagle Medium (DMEM) (Gibco) and fetal bovine serum (FBS) were applied for the following experiments. HaCat cells were cultured in DMEM supplemented with 10% (v/v) FBS and 1% penicillin. The cells were incubated in a humidified atmosphere with 5% CO_2_ at 37 °C. CCK8 assay. HaCat cells (1 × 10^4^ cells per well) were seeded in 96-well plates with 100 μL DMEM and incubated for 24 h before the tests. hPG-Gs, hPGN-Gs and hPGS-Gs in the culture medium were added to the medium-removed 96-well plates with different concentrations and incubated for another 24 h, respectively. After that, the culture medium solutions were removed and the cells were rinsed with PBS twice. 100 μL culture medium with 10 μL CCK8 solution was added to each well. After incubation for the following 2 hours, 75 μL medium was carefully transferred to a new plate to avoid the influence of graphene on final results, and the absorption was measured at wavelength of 450 nm with a microplate reader. Cells without any treatments were regarded as a negative control. Each sample was measured 3 times.

#### Cell uptake assay

The uptake of IDCC-labeled hPGN-Gs and hPGS-Gs by HaCat cells was analyzed using confocal laser scanning microscopy (CLSM). The cells were propagated as described above. For CLSM, HaCat cells were seeded in eight-well ibidi slides (ibidi treat) in 270 μL of DMEM. After cell attachment for 4 h to 24 h, 30 μL of a postseeding solution containing compound was added, and the cells were further incubated overnight. Before imaging, the cells were stained with Hoechst 33342 (1 μg mL^−1^), washed twice with PBS, and covered with fresh cell culture medium (DMEM). Confocal images were taken with an inverted confocal laser scanning microscope Leica DMI6000CSB SP8 (Leica, Wetzlar, Germany) with a 63×/1.4 HC PL APO CS2 oil immersion objective using the manufacturer-given LAS X software in sequential mode with the following channel settings: transmission Ch (gray intensity values), excitation laser line 405 nm, detection of transmitted light (photomultiplier); Ch1 (Hoechst 33342): excitation laser line 405 nm, detection range 410–484 nm (hybrid detector); Ch2: excitation laser line 488 nm, detection range 493–712 nm (hybrid detector).

#### Release of tacrolimus

hPGN-Gs and hPGS-Gs with 50 μg TAC content was dispersed separately in PBS (2 mL, pH 7.4), and equally transferred into 2 dialysis tubes (spectra/Por MWCO 2 kD). Then, the tubes were immersed in 2 vials containing 30 mL PBS (pH 7.4). Vials were set up in a thermostatic water bath (37 °C) on a heating rotator. 5 mL solution from PBS medium out of dialysis bag were collected at each 2 h intervals, and meanwhile, fresh PBS was replenished. One of the vials was irradiated with NIR laser (808 nm, 0.5 W cm^−2^) for 5 min every 2 h. The amount of released TAC at different time frames was determined by LC-MS/MS method. To this end, lyophilized samples were resuspended in acetonitrile containing 1 μM stable-isotopically labeled [^13^C_1_, d_4_]TAC. Samples were then shaken at 200 rpm for 1 h before particles were allowed to sediment for 1 h and supernatants were analyzed. The drug release studies were performed in triplicates.

#### Photothermal studies

hPGS-Gs and hPGN-Gs were dispersed in 2 mL PBS (100 μg mL^−1^ and 1000 μg mL^−1^) and then equally distributed into 2 tubes. Subsequently, near-infrared (NIR) laser irradiation (808 nm, 0.5 W cm^−2^) was performed to these tubes for 5 min. The temperature of the solutions in tubes were recorded by IR thermal imaging camera (FLIR, E40).

## Results and discussion

### Synthesis of hPGN-Gs, hPG-Gs and hPGS-Gs

**Fig. 1:**
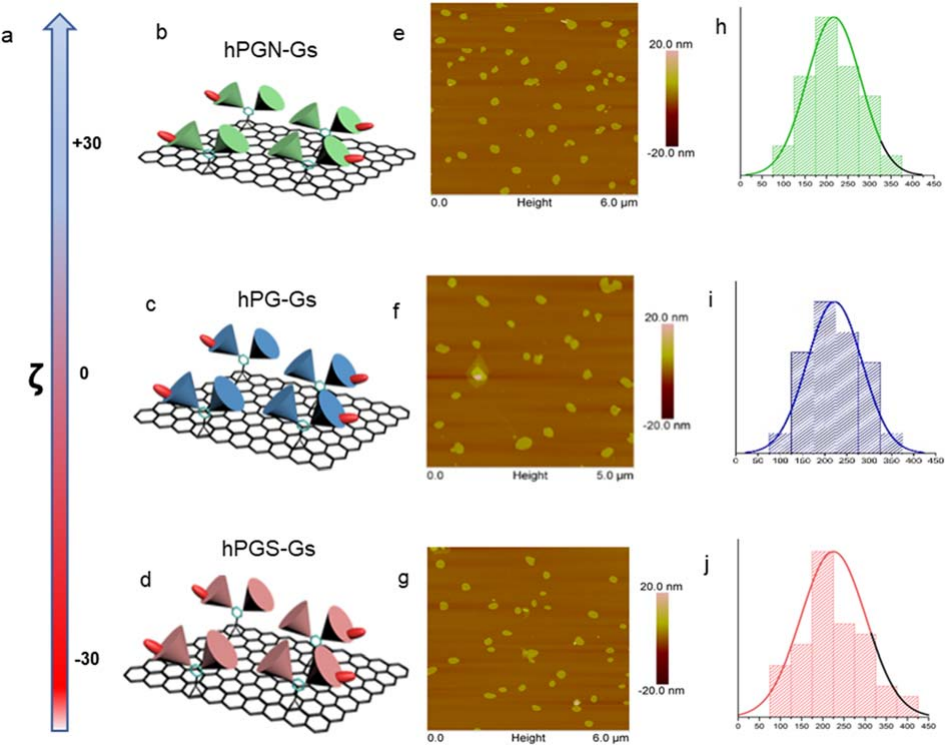
Characterizations of graphene sheets functionalized with polyglycerolamine (hPGN-Gs, top row), polyglycerol (hPG-Gs, middle row) and polyglycerol sulfate (hPGS-Gs, bottom row). (a) Surface charge of the functionalized graphene sheets (100 μg mL^−1^) in PBS. (b–d) Schematic representations of graphene sheets with polyglycerolamine (positive surface charge), polyglycerol (neutral) and polyglycerol sulfate (negative surface charge). (e–g) AFM images and (h–j) corresponding size profiles of graphene sheets with polyglycerolamine, polyglycerol and polyglycerol sulfate. Graphene sheets were functionalized with dichlorotriazine groups through nitrene [2 + 1] cycloaddition reaction, and polyglycerol (Mn = 10 000 g mol^−1^) with 7% amino functional groups was conjugated to their surface through nucleophilic substitution reaction. The obtained polyglycerol-functionalized graphene sheets (hPG-Gs), which were charge-neutral, were sulfated in accordance with our previously published methods, yielding their counterparts with sulfate functional groups and negative surface charge (hPGS-Gs).^46^ In order to synthesize graphene sheets with amino functional groups (hPGN-Gs) and positive surface charges, hPG-Gs were mesylated and then functionalized by ethylene diamine reagent.^46^ Zeta potential measurements showed respective surface charges of around +30 mV, 0 mV and −30 mV for hPGN-Gs, hPG-Gs, and hPGS-Gs (a–d). AFM images of the functionalized graphene sheets with different surface charges showed sheet like structures with 5.5 nm height and 200 nm average lateral size (e–g). The height of the functionalized sheets corresponded to a monolayer graphene sheet with polyglycerols conjugated to both of its faces. IDCC was conjugated to the sheets to track them after their uptake by HaCat cells or after penetration into human skin.^41^ The functionalized sheets' similar lateral sizes, polyglycerol content, and fluorescence intensity provided an opportunity for a comparative study on the effect of their surface charge on their skin penetration.

A series of polyglycerol-functionalized graphene sheets with 200 nm lateral sizes and different surface charges were synthesized according to our published procedures^39^ and characterized by means of atomic force microscopy and dynamic light scattering measurements (Fig. 1).

### Cytotoxicity and cellular uptake studies

**Fig. 2:**
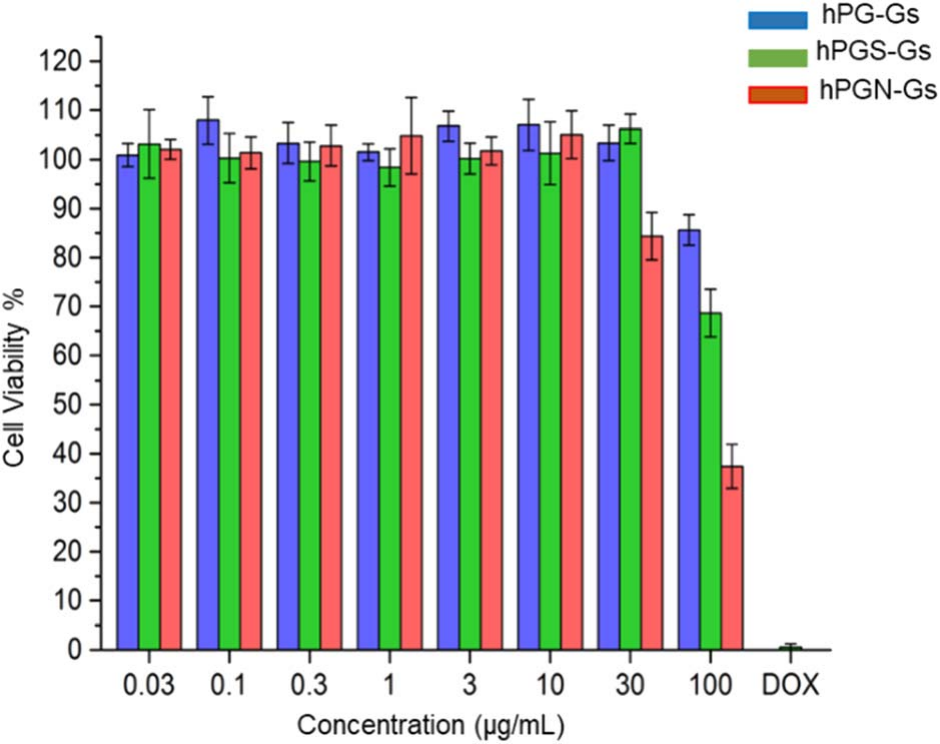
The CCK-8 assay for the HaCat cells after 24 h treatment with hPG-Gs, hPGN-Gs and hPGS-Gs. The untreated cells were considered our negative control, and the cells treated with doxorubicin hydrochloride (DOX·HCl) (2 μg mL^−1^ and 50 μg mL^−1^).

**Fig. 3:**
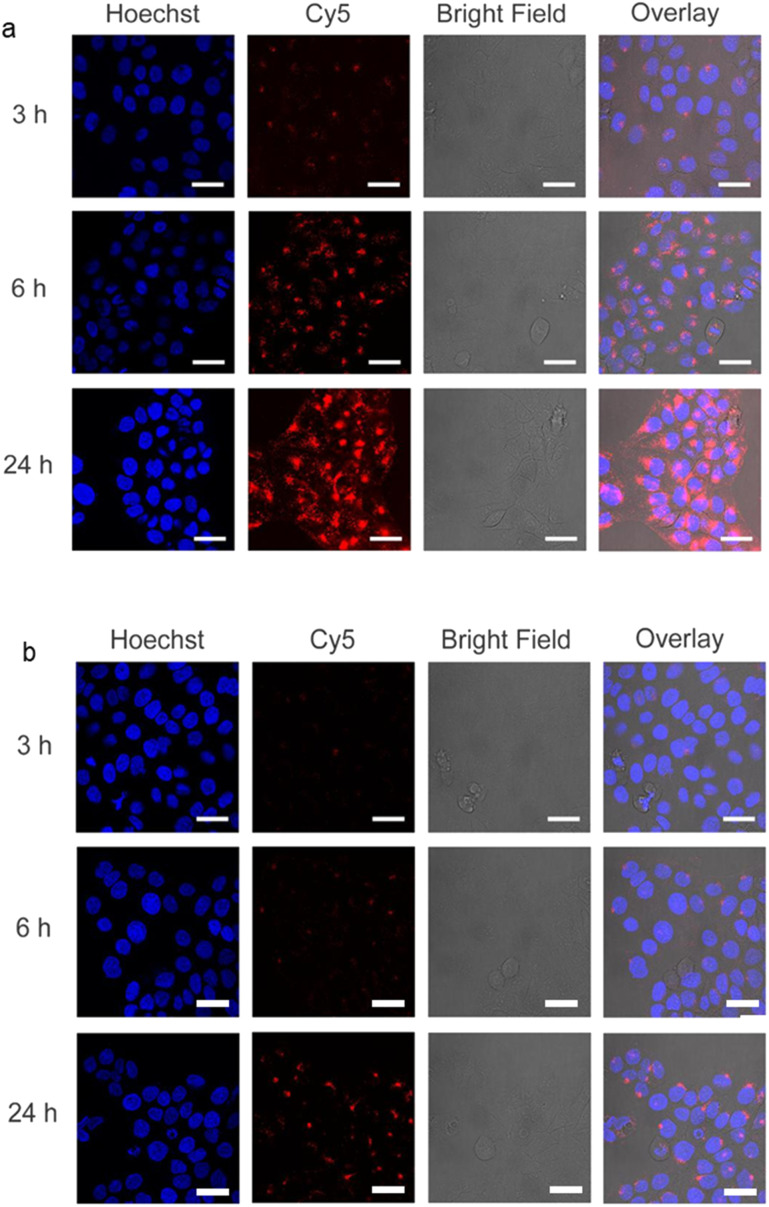
Confocal laser scanning microscopy (CLSM) images of living HaCat cells incubated with (a) IDCC-labeled hPGN-Gs at 10 μg mL^−1^ and (b) IDCC-labeled hPGS-Gs, also at 10 μg mL^−1^. Scale bars correspond to 50 μm.

In order to study the short-term biocompatibility of the functionalized graphene sheets, they were incubated with HaCat cells for 24 h, and the viability of these cells was investigated by CCK-8 assay (Fig. 2). None of the functionalized graphene sheets showed significant toxicity at concentrations of up to 10 μg mL^−1^. However, hPGN-Gs showed considerable toxicity at a concentration of 30 μg mL^−1^, as did the two other adducts (hPG-Gs and hPGS-Gs) at 100 μg mL^−1^. Afterwards, the uptake of the functionalized graphene sheets, hPGN-Gs and hPGS-Gs, by HaCat cells at different time frames was monitored. According to our previous study, hPG-Gs showed no significant uptake due to the polyglycerol coverage.^40–43^ While the negatively charged counterparts with sulfate functional groups showed considerable uptake after 24 h (Fig. 3a and b). However, hPGN-Gs showed the highest uptake efficiency, and their fluorescence signal after three hours' incubation was easily detectable inside the cells. Due to their large size, the graphene sheets stayed in the cytoplasm around the cell nucleus and did not enter this cell organelle. Although the membrane of cells is much thinner than skin layers, the efficient cellular uptake of graphene derivatives by HaCat cells indicated that graphene derivative may be internalized into keratinocytes resulting in deeper penetration in the viable epidermis. The IDCC-labeled used as positive controls. Functionalized graphene sheets were then subjected to interaction with human skin.

### Skin interaction studies of hPGN-Gs hPG-Gs and hPGS-Gs

**Fig. 4:**
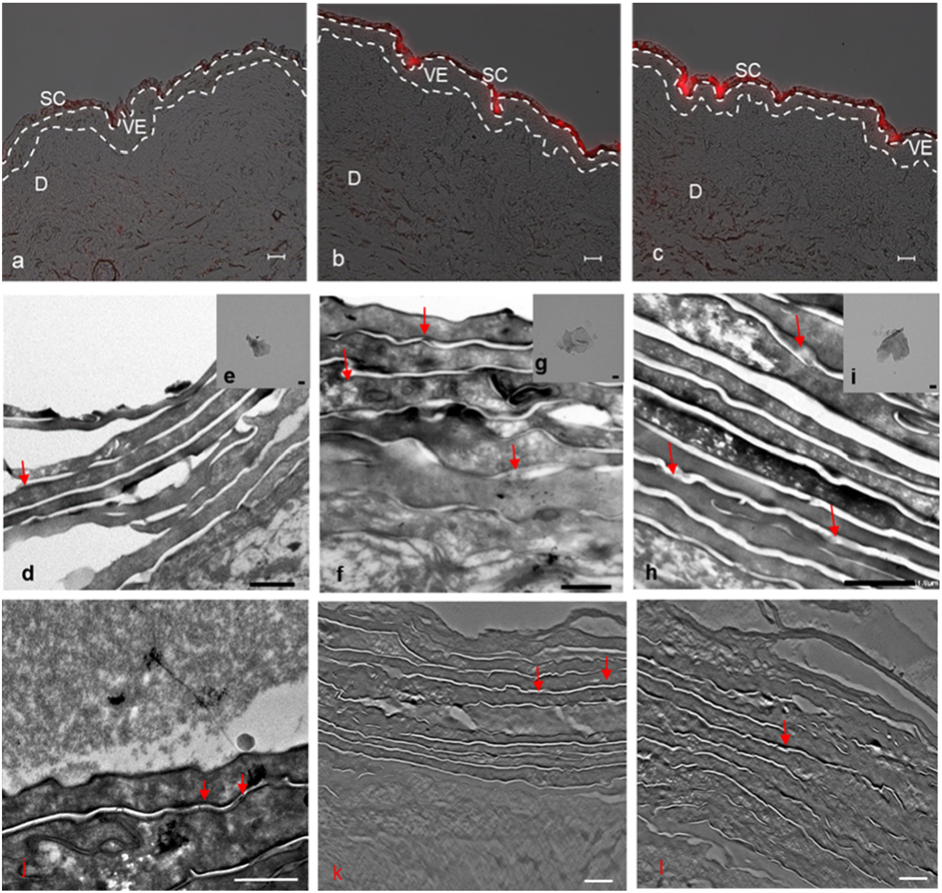
Representative overlay microscopy images (bright field and fluorescence) of human skin after 18 h topical application of graphene sheets: IDCC-labeled (a) hPG-Gs, (b) hPGN-Gs and (c) hPGS-Gs. Scale bars correspond to 50 μm. TEM images from ultrathin sections of human skin upon incubation with (d) hPG-Gs; (f) hPGN-Gs; and (h) hPGS-Gs. Assumed graphene sheets (indicated by red arrows) were found in the stratum corneum. Scale bars correspond to 1 μm. TEM images of functionalized graphene sheets namely (e) hPG-Gs; (g) hPGN-Gs; and (i) hPGS-Gs. Scale bars correspond to 100 nm. Images taken from electron tomography volume reconstructions, where graphene sheets are localized, (j and k) incubation with hPGN-Gs. Scale bars correspond to 1 μm and 2 μm, respectively, and (l) incubation with hPGS-Gs. Scale bar corresponds to 2 μm. SC: stratum corneum. VE: viable epidermis. D: dermis.

Skin penetration experiments and fluorescence microscopy images, using a conjugated IDCC signal, didn't show a significant penetration for hPG-Gs after 18 h incubation. This observation was in agreement with other reported results concerning low interaction between neutral graphene-functionalized nanomaterials and biosystems (Fig. 4a).^39,40^ However, hPG-Gs analogs with sulfate and amine functional groups interacted significantly with the skin (Fig. 4b and c). While both hPGN-Gs and hPGS-Gs predominantly remained in the stratum corneum layer, hPGN-Gs with the positive surface charge showed the most efficient skin interaction (Fig. S1† indicated untreated control skin image). These results were consistent with those from the cellular uptake studies using HaCat cells and are a warning signal of potential health concerns. But they may also point to an efficient strategy for topical delivery of therapeutic agents. Localizing the functionalized graphene sheets in the stratum corneum diminished the risk of their internalization into the human body and thus their interaction with inner organs.

To further study the interaction of graphene sheets with human skin, TEM images of the skin after topical treatment with hPG-Gs, hPGN-Gs and hPGS-Gs were recorded (Fig. 4d, 3f and h). TEM images of graphene sheets showed an average lateral size of 200 nm depending on their folding (Fig. 4e, g and i). The overview of the stratum corneum layer of the skin after 18 h of treatment suggests distribution of graphene sheets in this layer; this effect is more pronounced for the hPGN-Gs and hPGS-Gs samples than for the others (untreated control skin indicated in Fig. S2†). According to these results, functionalization of graphene sheets with positively charged amino or negatively charged sulfate groups increased diffusion of these materials across the corneocyte cell layers of the stratum corneum, while this effect was even less recorded for the hPG-Gs treated samples. Moreover, skin samples treated with hPGN-Gs and hPGS-Gs were further analyzed by transmission electron tomography imaging. This result further confirmed the assumption of internalization of hPGN-Gs and hPGS-Gs into the stratum corneum (Fig. 4j, k and l). Accordingly, hPGN-Gs and hPGS-Gs was further studied for their dermal drug delivery potential.

### Photothermal property of hPGN-Gs and hPGS-Gs

**Fig. 5:**
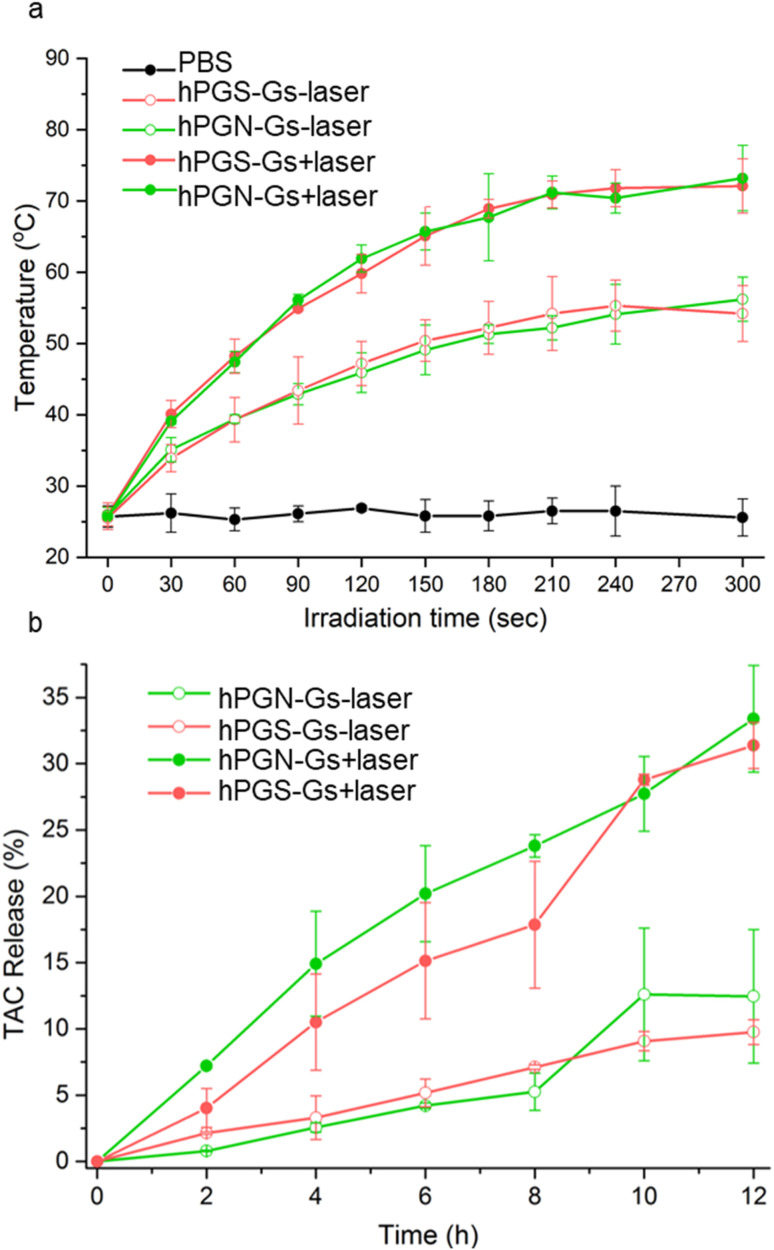
(a) Heat-generation kinetics of functionalized graphene sheets with different surface charges dispersed in PBS at different concentrations and under NIR irradiation (808 nm, 0.5 W cm^−2^). Pure PBS was used as control. (b) Tacrolimus release profile with (+laser) and without laser (−laser) irradiation.

The functionalized graphene sheets showed high photothermal conversion, which is an efficient stimulus factor for triggering the release of their cargo. Laser irradiation of an aqueous solution of hPGN-Gs and hPGS-Gs (1 mg mL^−1^) for 3 minutes increased their temperature to 65 °C (Fig. 5a). The photothermal conversion was concentration-dependent, and the temperature of a 100 μg mL^−1^ aqueous solution of these materials reached 50 °C after four minutes of laser irradiation.

### Drug encapsulation and release studies

Another advantage of using the functionalized graphene sheets as drug delivery systems is that they have shown a high loading capacity for hydrophobic therapeutic agents.^41^ Accordingly, we theorized that hPGN-Gs and hPGS-Gs that showed efficient skin penetration could be used as dermal delivery systems. They were loaded with tacrolimus (TAC), a topical therapeutic agent for treating T cell-mediated diseases such as eczema and psoriasis. TEM images of hPGN-Gs and hPGS-Gs after loading with tacrolimus showed sheets with comparable dimensions as before drug loading (Fig. S3†). hPGN-Gs and hPGS-Gs respectively showed 50% and 49% loading capacity for this drug. These capacities are much higher than those reported for other delivery systems such as polymeric nanocarriers^54,56^ and they make hPGN-Gs and hPGS-Gs worthwhile for investigation as dermal delivery systems. The release of TAC from the functionalized graphene sheets was investigated with and without laser irradiation. It was found that laser irradiation dramatically accelerated the rate of release of TAC from graphene sheets (Fig. 4b).

### Skin penetration studies of Nile red using NIR irradiation

**Fig. 6:**
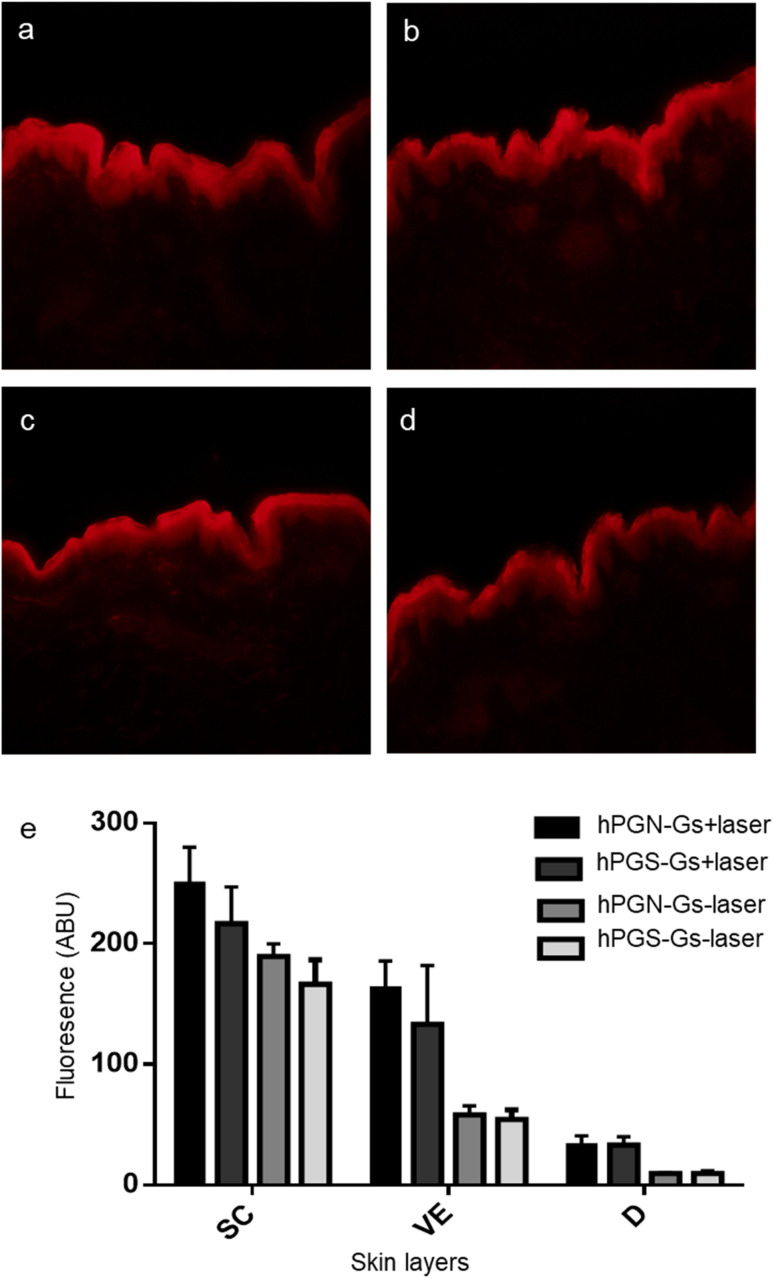
Representative fluorescence microscopy images of human skin after 18 h topical application of (a) hPGN-Gs and (b) hPGS-Gs loaded with Nile red without laser irradiation (−laser). (c) hPGN-Gs and (d) hPGS-Gs loaded with Nile red after laser irradiation (+laser). (e) Fluorescence intensity of Nile red in different layers of human skin treated with Nile red-loaded hPGN-Gs and hPGS-Gs with and without laser irradiation after 18 h incubation time and 3 treatments (*n* = 3, mean ± SEM). Scale bar corresponds to 50 μm.

Since TAC does not show a fluorescence signal, hPGN-Gs and hPGS-Gs were loaded with Nile red, and their ability to transfer and release of this molecule in different layers of skin was studied using fluorescence microscopy. hPGN-Gs and hPGS-Gs were able to significantly transport the loaded Nile red through the viable epidermis and dermis layers of skin (Fig. 6a and b). Moreover, skin irradiation by near-infrared (NIR) laser, after 6 h treatment with the Nile red-loaded hPGN-Gs and hPGS-Gs, efficiently increased release and penetration of the dye inside these two layers of skin was (Fig. 6c and d). The fluorescence signal intensity of Nile red loaded on hPGN-Gs and hPGS-Gs in different layers of skin was evaluated using fluorescence microscopy and by image analysis using ImageJ software. Upon laser irradiation, hPGN-Gs showed higher Nile red penetration into the skin layers more than hPGS-Gs. In this study, after 6 h of treatment with hPGN-Gs, we applied laser irradiation intermittently over 30 min for a total exposure time of 2.5 min. As it was expected, after this irradiation treatment, penetration of Nile red increased 2.2-fold in the viable epidermis and threefold in the dermis. Under the same conditions, after laser irradiation and 18 h treatment with hPGS-Gs, the release of Nile red increased 1.5-fold in the viable epidermis and threefold in the dermis (Fig. 6e). These results confirmed that a therapeutic agent can be efficiently transported through the skin by hPGN-Gs and hPGS-Gs, and that the release of this cargo can be accelerated using the photothermal property of graphene sheets.

## Conclusion

This study evaluates possible interactions between of functionalized graphene sheets with lateral size 200 nm and different surface charge – hPGN-Gs (+30 mV), hPG-Gs (0 mV) and hPGS-Gs (−30 mV) – and human skin. As a first step, we evaluated cytotoxicity and cellular uptake of these materials against HaCat cells for 24 h incubation time. The results demonstrate that hPGN-Gs, hPG-Gs and hPGS-Gs do not show significant toxicity up to 30 μg mL^−1^, while a concentration increase of hPGN-Gs and hPGS-Gs to 100 μg mL^−1^ respectively, leads to toxic effects for HaCat cells. Cellular uptake studies indicate that hPGN-Gs and hPGS-Gs are efficiently internalized into the cell cytoplasm at a concentration of 10 μg mL^−1^. Skin penetration experiments followed by fluorescence microscopy and TEM observations confirm internalization of hPGN-Gs and hPGS-Gs into the top layer of human skin, the stratum corneum, after 18 h of exposure time, whereas hPG-Gs shows weak skin uptake. These observations led us to further investigate hPGN-Gs and hPGS-Gs for their potential use in topical drug delivery. hPGN-Gs and hPGS-Gs show a high drug-loading capacity (∼50 w/w%) for tacrolimus, and they were able to release the drug under short-term NIR laser irradiation. In addition, skin penetration experiments of Nile red-loaded hPGN-Gs and hPGS-Gs confirmed that NIR irradiation increases the release of Nile red inside the skin. In particular, hPGN-Gs enhanced the Nile red's penetration in the viable skin layers more efficiently than hPGS-Gs. Therefore, the potential health risk of graphene should be considered and investigated further, *e.g.* in *in vivo* experiments. Outcomes of this work shed a light in the future biomedical applications and toxicity of similar carbon based nanomaterials including fullerene, carbon nanotubes and graphitic materials upon exposure to human skin.

## Author contributions

Conceptualization, methodology, *ex vivo* skin studies, data interpretation, writing—original draft preparation, visualization, F. Z.; synthesis, *in vitro* cell studies, drug loading and release studies Z. T.; LC-MS/MS measurements, data interpretation, visualization, methodology, F. S.; TEM imaging from graphene sheets, data interpretation, visualization, K. L.; TEM imaging from skin samples, data interpretation, visualization, S. K. and C. B.; *ex vivo* studies, data interpretation, visualization, F. R.; conceptualization, guidance, resources, funding acquisition, B. K.; J. P.; S. H.; A. V. and R. H. All authors have read and agreed to the published version of the manuscript.

## Conflicts of interest

There are no conflicts to declare.

## Supplementary Material

NA-005-D3NA00574G-s001
