# Supplementary material for: Efficient skin interactions of graphene derivatives: challenge, opportunity or both?
Source: Nanoscale Adv. 2023 Oct 11;5(21):5923–31. doi: 10.1039/d3na00574g (PMC10597544; doi:10.1039/d3na00574g)
Supplement: NA-005-D3NA00574G-s001 [file NA-005-D3NA00574G-s001.pdf]

Electronic Supplementary Material (ESI) for Nanoscale Advances.
This journal is © The Royal Society of Chemistry 2023

Efficient Skin Interactions of Graphene Derivatives: Challenge, Opportunity or both?

Fatemeh Zabihi*[a,b], Zhaoxu Tu[a,c], Sabine Kaessmeyer[d,i], Fabian Schumacher[e], Fiorenza Rancan[b], Burkhard Kleuser[e], Christoph Boettcher[f], Kai Ludwig[f], Johanna Plendl[d], Sarah Hedtrich[g,h], Annika Vogt[b], Rainer Haag*[a]

a Institut für Chemie und Biochemie, Freie Universität Berlin, Takustr. 3, Berlin 14195, Germany

b Clinical Research Center for Hair and Skin Science, Department of Dermatology and Allergy, Charité Univer-sitaetsmedizin Berlin

c the Sixth Affiliated Hospital of Sun Yat-sen University, Guangzhou, Guangdong, China

d Institute of Veterinary Anatomy, Department of Veterinary Medicine, Freie Universität Berlin, Germany

e Institute of Pharmacy (Pharmacology and Toxicology), Freie Universität Berlin, 14195 Berlin, Germany

f Forschungszentrum für Elektronenmikroskopie, Institut für Chemie und Biochemie, Freie Universität Berlin, Fabeckstr. 36a, 14195 Berlin, Germany

g University of British Columbia, Faculty of Pharmaceutical Sciences, 2405 Wesbrook Mall, V6T1Z3 Vancouver, Canada

h Berlin Institute of hHealth, at Charité Universitaetsmedizin Berlin, Lindenberger Weg 80, 13125 Berlin, Ger-many

i Division of Veterinary Anatomy, Vetsuisse Faculty, University of Bern, 3012 Bern, Switzerland

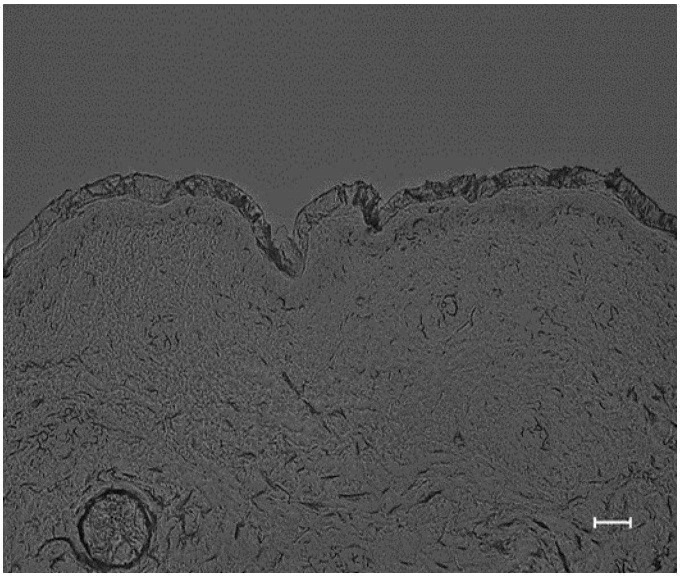

Figure S1: Representative overlay microscopy images (bright field and fluorescence) of untreated human skin. Scale bars correspond to 50 µm.

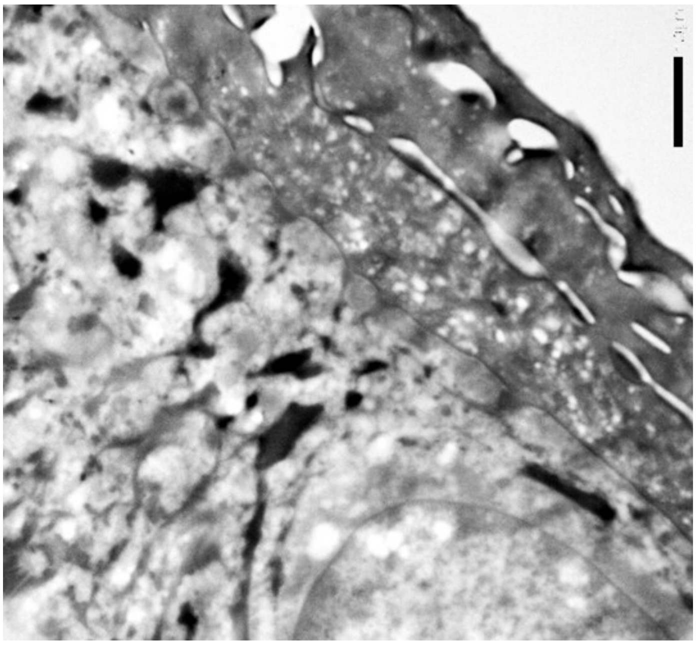

Figure S2: Transmission electron microscopy images of untreated human skin. Scale bars correspond to 1 µm.

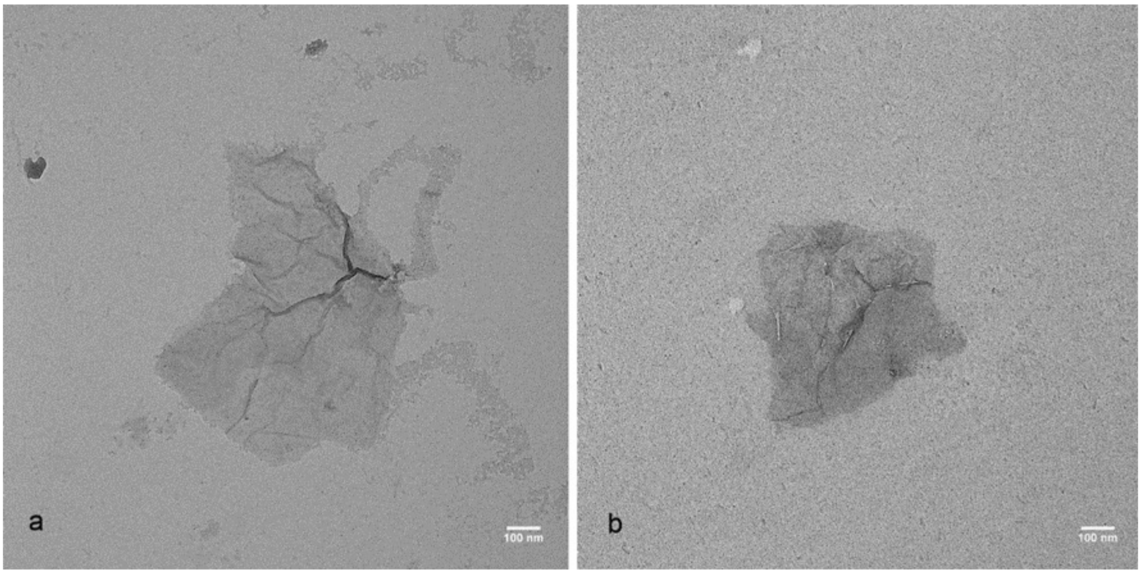

Figure S3: Transmission electron microscopy images of (a) hPG-amine covered TRGO (hPGN-Gs) and (b) hPG-sulfate covered TRGO (hPGS-Gs), each loaded with the drug tacrolimus, Scale bars correspond to 100 nm.
